# The Boarding out of Pauper Children

**Published:** 1889-08-31

**Authors:** 


### The Boarding Out of Pauper Children.

### I.—THE SYSTEM ITSELF.

For almost exactly twenty years past the practice of boarding out pauper children has been tolerated and sanctioned, if not positively encouraged, by the Government department charged with the administration of "the poor laws." The probationary period is now closed, and the principle and practice of boarding out are formally accepted in Local Government Board Orders of the present year, containing new and comprehensive regulations upon the whole system. It has been, unquestionably, a wise decision to appoint a competent lady as an inspector of the homes where the children are lodged, and of all matters bearing upon their maintenance, training, health, cleanliness, etc. Miss M. H. Mason's report is one of the most interesting official documents we have ever studied, and we cannot doubt that many of the new rules are based upon the ample and detailed information which she was able to place at the service of the Local Government Board.

The opponents of the boarding-out system, and even the strongest advocates of it, are fully conscious of one special danger attending it. "If the boarding-out system spreads widely," says Miss Mason, "only strict rules can save it from degenerating into baby-farming." Some instances are given in illustration of this special need of watchfulness against a most possible and quite fatal abuse of what is in itself a humane and charitable plan: "I found two boys boarded out with an elderly woman, who said that having been left a widow, she had taken in children as a means of support for herself and a daughter too delicate for service. She had, besides the two boys whom I came to see, two others placed there by a lady not on the committee, another, who, I was told, was the child of a gentleman, two girls from some other source, and others for whom there was not room in the house, and who slept out, boarding here by day. I saw six children in the house, and the woman refused insolently to tell me how many more there were. She had, since the children had been placed here, married a cripple, whom I saw on his crutches; and this family of three persons were supported on their payments."

Other instances are given of eight children boarded in one house, and six in another. The obvious inference is that, in cases of this kind, a livelihood is made entirely out of the small sums paid for the maintenance of the children, with the certainty of cruel injustice to them. If a child, immediately dinner is over, is found greedily devouring a piece of mouldy bread which comes in his way, he is evidently lodged in an unsuitable home, and with unsuitable foster-parents. On this head, homes may be unsuitable in two ways. Foster-parents may starve a child merely to make a profit, and because they care nothing about him. They may also do it because, whilst really caring for the child, they are themselves too poor to provide sufficient food for their households.

We have purposely set out at first the real abuses which have to be guarded against, and which are, we notice with pleasure, fully and completely guarded against in the new rules and regulations of the Local Government Board. Like Miss Mason herself, we would conceal none of the dangers which accompany the boarding-out system, whilst, like this competent authority, we are also entirely persuaded of the excellence and real charity of the plan when administered under proper supervision and legal control.

We might have begun with the merits of the system, the lief-long benefits it usually confers upon those pauper children for whom it is intended, and the pleasure the children themselves find it to have a real home, foster-parents who love them, and domestic occupations in which they interest themselves just like other children, never driven to seek the shelter of a workhouse. Every year, many thousands of children are born in our workhouses. There is a worse misfortune, namely, to grow up amidst pauper surroundings, with the impression upon one's own mind for life of belonging to a marked and degraded class of the community. These somewhat inevitable evils of a great state charity are mitigated, indeed quite taken away, in the case of one large section of pauper children, by the system of boarding out. One is glad to notice also that voluntary benevolence now aims at befriending poor children in the same way. Dr. Barnardo has recourse to it for some of the many orphans committed to his care, and has secured the services of a lady doctor for regular and complete examination of the children placed here and there in country homes. The Church of England Society for Providing Homes for Waifs and Strays likewise favours this system, either because its own admirably managed homes are full, or because of the advantages some children are sure to gain from being brought up in what will later be their natural surroundings. We are not aware what steps are taken by this society against possible abuses of their charity and injury to the children, but our knowledge of its general management assures us that a matter of so much moment has received due attention. We can wish no more than that all boarded-out children should be as comfortable and happy as those "deserted and orphan children" sent to country homes by the guardians of the poor.

When visiting homes, foster-parents, and children in the neighbourhood of Windermere, Miss Mason tells us:

"I met a girl whom I had already visited walking with another, whom I did not as yet know. The one whom I did not know smiled at me as if she had an equal claim on my attention. I asked her name, and she answered in the most cheerful of tones, 'I am an orphan too,' as if to be an orphan were the most enviable of positions, and the key to all privileges."

But there is a record from Campsea Ash (Suffolk) which deserves to be given fully, and may be profitably studied by any who are opposed to the boarding out of pauper children.

"Three children—a girl of ten, and two boys of three and four—were placed together, contrary to the boarding-out order, none of them being brothers and sisters. Their foster-mother was an unmarried woman, keeping house for her father, a carpenter. The youngest was now a very fine child, but the deep scars left by abscesses on his neck proved the truth of what I was told as to his extreme delicacy when he first came, and that he had recovered owing to her great care. I asked her if she should be obliged by the regulations to part with one of the children which it should be. She answered that nothing could induce her to part with the youngest boy; that 'Jenny' was such a good girl that she should not like to lose her, and that her father was too fond of her to let her go; and that therefore it would have to be 'Tommy,' but she hoped that he might not be taken from her, because his complaints and habits were so troublesome and repulsive that she feared his not being treated with care and patience elsewhere. This child had been placed here by the committee members because no one else would have him. He was said to be naturally very unhealthy, and his appearance fully confirmed it. His nose had been broken before he came here, and his front upper teeth knocked out. He had a discharge from the ears and nose, and had had a bad head, now nearly cured. His head was mis-shapen, he was rather deaf, could not speak intelligibly, did not seem of full intellect, and was very dirty in his habits. These were the reasons which this woman gave for wishing to keep him; and I was told by others that he had arrived in a still worse condition, and had much improved, thanks to her unwearying care."

Poor little Lazarus, happy in being laid at the *poor* man's gate.

